# A Modified Microscopic-Endoscopic Bilateral Transseptal Approach for Pituitary Adenomas: Comparisons of Nasal Outcome and Quality of Life Using the Microscopic Transnasal Approach

**DOI:** 10.3389/fonc.2022.778704

**Published:** 2022-02-08

**Authors:** Junjie Zhong, Yanfang Gu, Jie Zheng, Bojie Yang, Zengxin Qi, Tianwen Li, Chao Shen, Zhifeng Shi

**Affiliations:** ^1^ Department of Neurosurgery, Huashan Hospital, Shanghai Medical College, Fudan University, Shanghai, China; ^2^ Neurosurgical Institute of Fudan University, Shanghai, China; ^3^ Shanghai Clinical Medical Center of Neurosurgery, Shanghai, China; ^4^ Shanghai Key Laboratory of Brain Function Restoration and Neural Regeneration, Shanghai, China; ^5^ National Center for Neurological Disorders, Shanghai, China; ^6^ Research Units of New Technologies of Micro-Endoscopy Combination in Skull Base Surgery (2018RU008), Chinese Academy of Medical Sciences, Shanghai, China

**Keywords:** olfaction, smell, anterior skull base, pituitary, endoscopic skull base, trans-transseptal, trans-sphenoid

## Abstract

**Objectives:**

In this study, we introduced a novel modified microscopic-endoscopic bilateral transseptal approach for pituitary adenoma resection to minimize surgery-related nasal injury. We also retrospectively compared comprehensive nasal outcomes and quality of life between the microscopic transnasal approaches.

**Methods:**

Patients with pituitary adenomas who underwent modified microscopic-endoscopic bilateral transseptal or microscopic transnasal approaches were assessed for olfactory function and quality of life using the Sniffin’ Sticks test, the Sino-Nasal Outcome Test-22 (SNOT-22), the SF-36, the anterior skull base (ASK) nasal inventory, and the subjective visual analog scale (VAS) before and 1 and 3 months after surgery. A nasal endoscopy procedure was also performed to evaluate structure abnormalities at 1 and 3 months after surgery.

**Results:**

Fifty-eight patients who underwent either modified microscopic-endoscopic bilateral transseptal (35 patients) or microscopic transnasal (23 patients) surgery were consecutively enrolled. Patients who underwent either transnasal approach experienced similar surgical complications, except for intraoperative cerebrospinal fluid leakage (43.5% vs 14.3% for modified microscopic-endoscopic bilateral transseptal or microscopic transnasal approach, respectively; *p* = 0.013). Patients who underwent the two approaches fully recovered according to the SF-36, SNOT-22, VAS, and Sniffin’ Sticks surveys, but not ASK scores, 3 months post-operatively. There was no significant difference in nasal endoscopy outcome at 3 months follow-up between the two approaches.

**Conclusions:**

The modified microscopic-endoscopic bilateral transseptal approach showed largely similar nasal mucosa protective outcomes to those of the microscopic transnasal approach for pituitary adenoma surgery. After pituitary adenoma resection using the modified approach, patients’ postoperative olfactory function, nasal structure, and quality of life can be restored to preoperative status within 3 months.

## 1 Introduction

Surgical approaches in the sellar region have evolved during the past century. In the 1897, Davide Giordano, a Venetian anatomist and surgeon, proposed a transglabellar approach to the pituitary involving resection of the nose and frontal sinus, followed by removal of the ethmoid bone, allowing wide access to the sphenoid sinus and sella (1). Hermann Schloffer was greatly inspired and extensive researched on the surgical methods of the pituitary gland, and reported the first successful removal of a pituitary tumor *via* a superior transsphenoidal approach in 1907. Combing previous investigations, Harvey Cushing, in 1910 to 1925, treated 231 pituitary tumors *via* the trans-sphenoidal route with a mortality rate of 5.6% in the preantibiotic era (2). Since the 1990s, endoscopic endonasal techniques have been integrated into sellar surgery because of developments in endoscopic concepts of nasal surgery and improvements in endoscopic equipment (3). As fully endoscopic trans-sphenoidal approach has been widely applied, great credit should be given to Edward Laws for having studied the technique and the pituitary adenoma in all its aspects, with a personal series of >6000 cases treated transsphenoidally. The endoscope provides a panoramic view of the suprasellar and parasellar compartments, especially with the use of an angled endoscope. This enhanced visualization has enabled improvements in the extent of resection and reductions in hospital stay duration and operative complications compared with those following the traditional microscopic transnasal transsphenoidal approach (4–7). However, nasal complications following full endoscopic approaches remain controversial. Several studies have reported that endoscopic surgery offers similar or perhaps more advantages over microscopic approaches for the protection of olfactory function (8–11), whereas other studies have reported the endoscopic approach changes the postoperative nasal anatomical structure and may result in headache, nasosinusitis, rhinorrhea, nasal incrustation, anosmia, and disturbance of ventilation (12–18). Theoretically, a binostril four-hand operation endoscopic surgery requires the removal of more nasal structures to make more room for endoscopic instruments. Therefore, maintaining postoperative nasal structure and function during binostril microscopic surgery is not as easy as during uninostril microscopic surgery. It has been reported that compared with the endoscopic approach, microscopic pituitary surgery provides better early postoperative sinonasal quality of life (QoL) and comparable olfactory outcomes (19). However, numerous modified endoscopic transnasal approaches have been developed to further protect the postoperative integrity of nasal function, such as the single-nostril transseptal transsphenoidal approach (20), the bilateral modified nasoseptal rescue flaps approach (21), the bilateral transseptal approach (with the help of a nasal speculum) (22), and the binostril approach (one side transseptal with the other side transnasal) (23). All approaches have shown good postoperative olfactory function and sinonasal QoL; however, few have been compared with typical uninostril transnasal microscopic approaches. Therefore, in this paper, we introduced a new modified microscopic-endoscopic bilateral transseptal approach without the use of a speculum and compared postoperative sinonasal QoL, QoL, olfactory function, and nasal structure integrity outcomes with those of the traditional microscopic trans-nasal approach for pituitary adenoma resection.

## 2 Materials and Methods

### 2.1 Patient Population and Study Design

We retrospectively reviewed all patients who underwent pituitary tumor resection using the modified microscopic-endoscopic bilateral transseptal approach (transseptal approach) and the microscopic transnasal approach (microscopic approach) by the same surgeon (B.Y.) at Huashan hospital between March 2019 and January 2021. The study was approved by the Institutional Ethics Committee of Huashan hospital Fudan University.

Patients (aged 18–65 years) underwent nasal endoscopy and magnetic resonance image (MRI) of the head before surgery, and those with a history of previous sinonasal surgery or nasal abnormalities were excluded. Patient diagnosed with neurodegenerative disease, which might cause olfactory disorder, were excluded. Patient demographics, tumor dimensions, length of hospital stay, surgical approach, outcomes, complications, and pathologic diagnosis (according to the World Health Organization classification) were collected. The goal of the surgery (gross total resection or subtotal/partial resection) was established preoperatively and determined by neuroradiologists according to the MRI obtained postoperatively as part of routine care.

### 2.2 Quality of Life, Olfactory Function, and Nasal Outcome Assessments

QoL was evaluated using the 36-item short-form health survey (SF-36). Olfactory function was assessed by the Sniffin’ Sticks test (Burghardt, Wedel, Germany) and the Visual Analogue Scale (VAS) according to the manufacturer’s protocols (24, 25). The Sniffin’ Sticks test has been confirmed to be suitable to be applied to a Chinese population (26). Examinations were performed by an independent person who was not involved in the surgical procedure. We only assessed binostril olfaction because some patients felt discomfort during the full test. The TDI score was calculated as the sum of odor threshold (OT), discrimination (OD), and identification (OI) scores. Because olfactory function varies by sex and age, the 10th percentile is routinely used to define the lower limit of normosmia in different age groups. A total TDI score (i.e., OT, OD, and OI) of ≤ 15 indicated that the patient was functionally anosmic. In cases whose TDI score was above the 10th percentile for their age group, the patient was considered normosmic, otherwise, hyposmia was considered. Nasal QoL and outcome were assessed subjectively *via* the 22-item Sino-Nasal Outcome Test (SNOT-22) (27) and the Anterior Skull Base Nasal Inventory-12 (ASK Nasal-12) (28). All tests and surveys were conducted before and 1 and 3 months after surgery. At postoperative follow-up, we examined patients using a nasal endoscope in the outpatient clinic of the otorhinolaryngology department and recorded nasal structural abnormalities, which included nasal septum perforation, nasal scabs, nasal congestion, nasal ostium obstruction, hypertrophy of the nasal turbinate, and deviated nasal septum.

### 2.3 Surgical Technique

All surgical procedures were performed by a senior neurosurgeon (B.Y.) with over 5 years’ experience in both microscopic and endoscopic transnasal pituitary surgery. Only endoscopic tumor resection requires two neurosurgeons, whereas other procedures can be completed by one neurosurgeon. Neuro-navigation was routinely used in tumor recurrent cases or those with other complicated conditions.

#### 2.3.1 Microscopic Transnasal Approach

After administration of general anesthesia, the patient was elevated by 15° using an operative pillow. The patient’s head was then fixed in a head holder with 10°–20° rotation towards the operator. A standard microscopic uninostril transseptal technique was performed during the surgery. After binostril preparation and draping, a hemitransfixion incision was made in the right nasal septal mucosa. Then, a subperiosteal dissection was performed between the septal bone and mucosa with a nasal speculum to expose the anterior wall of the sphenoidal sinus. We opened the sphenoidal sinus widely and resected the sphenoidal spectrum and sinus mucosa to reach the sellar floor. An X-shaped incision was used to incise the dura, and the tumor was resected using various angled curettes, pituitary rongeurs, and suction. Following the resection of the tumor and hemostasis, we carefully inspected the sellar cavity and used multilayer techniques to reconstruct the sellar floor, which included fat (if necessary), Dural Graft Matrix (DuraGen, INTEGRA, USA), artificial dura (Aesceulap, USA), and a Porcine Fibrin Sealant Kit (BeiXiu, China). Finally, we inspected the whole surgical cavity, repositioned the right septal mucosa flap, and inserted two nasal tamponades (MEROCEL, Medtronic, USA) into each nasal cavity.

#### 2.3.2 Modified Microscopic-Endoscopic Bilateral Transseptal Approach

##### 2.3.2.1 Microscopic Phase

Most procedures of this phase were the same as those of the microscopic transnasal approach. We removed the bony sphenoidal sinus under microscopy as much as possible to reach the same exposure extent as that of the endoscopic surgery to avoid the limitation of maneuverability of endoscopic instruments. After adequate removal of the bony sphenoidal sinus, spectrum, and sellar floor, we made another hemitransfixion incision in the left septal mucosa. Subperiosteal dissection was performed to complete the binostril septal surgical trajectory. Each septal mucosa flap was pulled laterally by one suture fixed to the surgical drapes to maintain the binostril septal entrance.

##### 2.3.2.2 Endoscopic Phase

The bilateral septal mucosa flaps were infiltrated by two gauze strips with lidocaine-containing epinephrine (1:100,000) solution for vasoconstriction. Each gauze strip was placed between the posterior nasal septum and septal mucosa flap on the bony surface, inferior to the anterior sphenoidal wall, to maintain the surgical trajectory.

**Figure 1:**
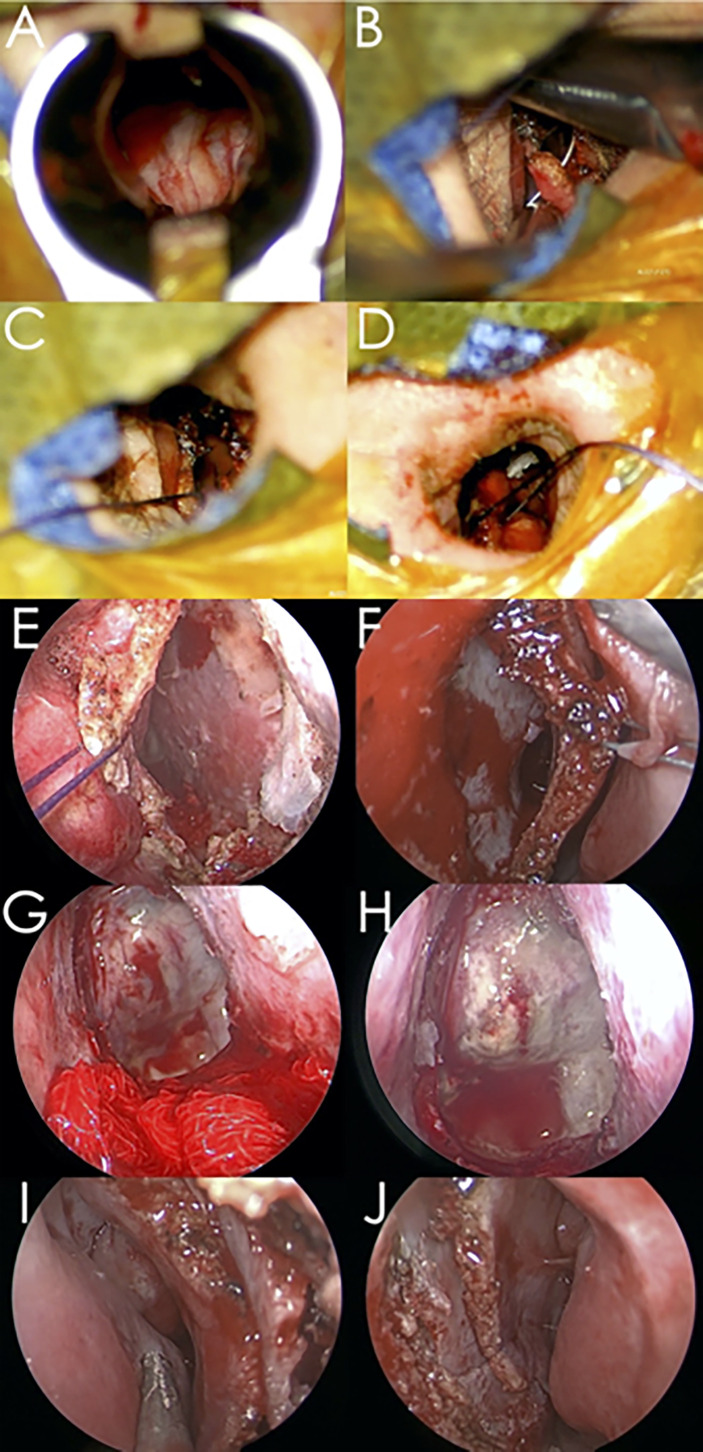
Representative surgical procedure images involved in the modified microscopic-endoscopic bilateral transseptal transsphenoidal approach. **(A)** Removal of the bony sellar floor using the uninostril microscopic technique. **(B)** Make a tagging suture on the left septal mucosa. **(C)** Suture between the left septal mucosa flap and the surgical drapes. **(D)** Suture between the right septal mucosa flap and surgical drapes. **(E)** Endoscopic view of the right nostril entrance. **(F)** Endoscopic view of the left nostril entrance. **(G)** Two epinephrine gauze strips were placed between the septal mucosa flaps to maintain the surgical corridor. **(H)** Endoscopic view of the seller floor and internal part of the olfactory cleft area mucosa. **(I)** Inspection of the right nasal cavity and repositioning of the septal mucosa flap. **(J)** Inspection of the left nasal cavity after final inspection. Asterisk: internal part of the olfactory cleft area mucosa.

After using a hydrogen peroxide cotton sponge to sterilize the surgical working field, the binostril endoscopic technique was performed for tumor resection (**Figure 1**). After resection and hemostasis of the sellar cavity, a multilayer technique was used to reconstruct the sellar floor. We then repositioned the two nostril septal flaps, inspected the two nasal cavities, and inserted two nasal tamponades.

### 2.4 Statistical Analysis

Data were analyzed using standard software (jamovi) with Student’s t-test, Wilcoxon-signed rank test, and paired t-tests. Statistical significance was set at *p* < 0.05.

## 3 Results

### 3.1 Patient Characteristics

**Figure 2:**
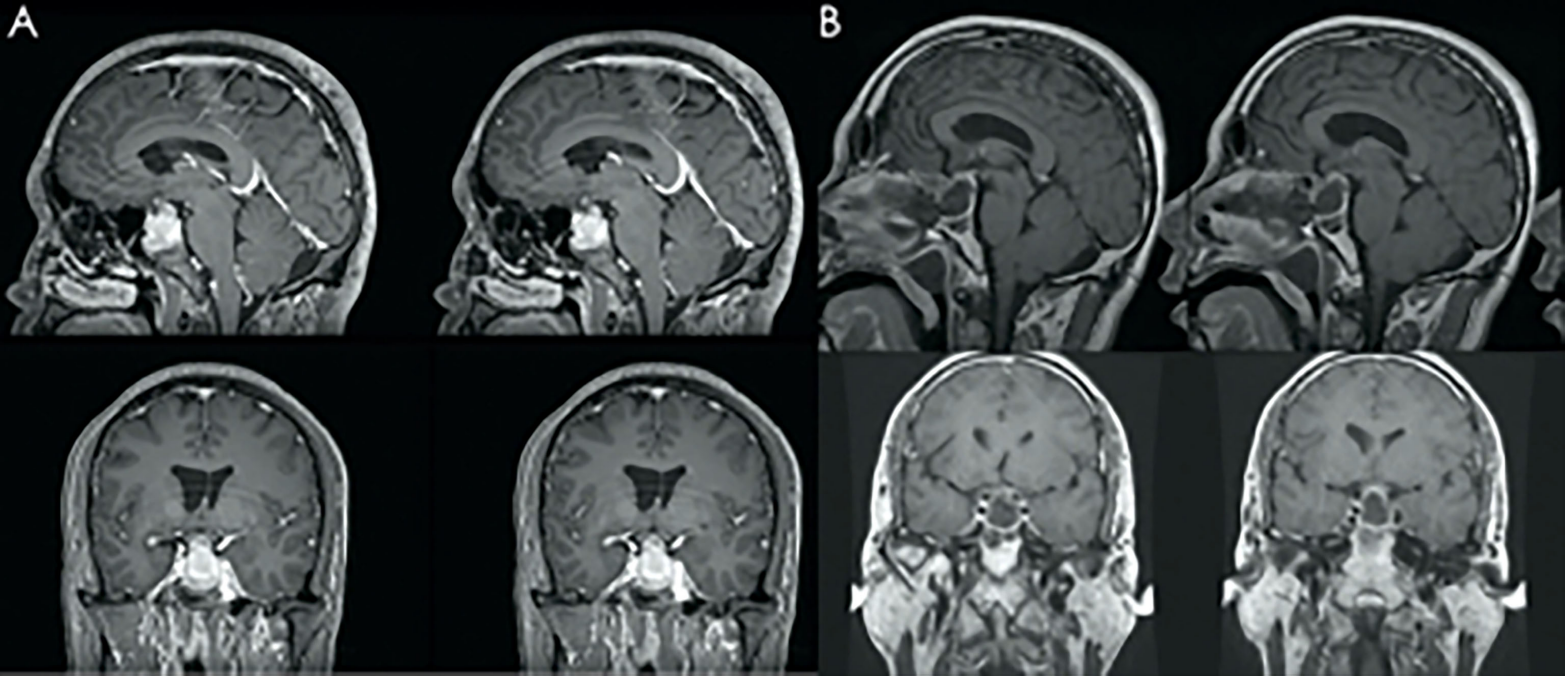
Representative image of preoperative magnetic resonance imaging (MRI) **(A)** and postoperative MRI follow-up **(B)**. The MRI shows gross total resection of the tumor.

Fifty-eight patients were enrolled in this study. Thirty-five patients underwent the microscopic approach, and 23 underwent the transseptal approach. There were no significant differences in age, sex, length of hospital stay, or Knosp grade between the two approach groups (**Table 1**). However, the tumor volume of the transseptal group was significantly larger compared with that of the microscopic group (10.5 ± 16.3cm vs 2.90 ± 4.36cm; *p* = 0.011). In the microscopic group, gross total resection (GTR) of the tumor was achieved in 30/35 patients, and GTR of the tumor was achieved in 20/23 patients who underwent the transseptal approach. GTR was not significantly different between the two groups. **Figure 2** shows the representative preoperative and postoperative magnetic resonance images of the two groups.

**Table 1:** Patient demographics.

Variable	Microscopic (n = 35)	Transseptal (n = 23)	*P* Value
Age in yrs	37.8 ± 10.9	41.7 ± 10.4	0.180
Sex			0.200
Male	16	7	
Female	19	16	
Length of stay in days	4.49 ± 1.69	5.09 ± 1.38	0.160
Functional adenomas	24	17	0.311
GH-secreting	3	3	0.584
luteinizing hormone/follicle stimulating hormone secreting	4	4	0.519
Prolactinoma	9	5	0.729
Adrenocorticotropic hormone secreting	3	2	0.987
Plurihormonal	5	3	0.893
Tumor vol in cm^3^	2.90 ± 4.36	10.53 ± 16.34	**0.011**
Knosp grade		
1-2	12	8	0.970
3-4	23	15
Gross total resection	30	20	0.893

Values represent mean ± standard deviation.

Bold values: statistically significant.

### 3.2 Quality of Life Scales

QoL and SF-36 scale scores before and 1 and 3 months after surgery are provided in **Table 2**. Although preoperative bodily pain (BP) and general health perception (GH) subdomain scores were lower in the transseptal group than in the microscopic group, there were no significant differences in the SF-36 score in either group at 1 or 3 months postoperatively. In the microscopic group, Physical (PF) and physical role (PR) functioning subdomain scores showed significant decreases 1 month postoperatively (*p* = 0.005 and *p* < 0.001, Wilcoxon-signed rank test) and full recovery 3 months postoperatively from pre-operative scores (*p* = 0.178 and *p* = 0.906, respectively, Wilcoxon-signed rank test). However, other subdomains did not significantly change 1 or 3 months postoperatively. In the transseptal group, the PF and PR subdomain scores significantly decreased at 1 month postoperation (*p* = 0.004, *p* = 0.002, respectively, Wilcoxon-signed rank test). However, only the PR score fully recovered 3 months postoperatively from preoperative scores (*p* = 0.105, Wilcoxon-signed rank test), whereas the PF subdomain score did not (*p* = 0.019, Wilcoxon-signed rank test). The GH subdomain score significantly improved at both 1 and 3 months postoperatively.

**Table 2:** SF-36.

	Preoperative			PO 1 Month			PO 3 Months		
	Microscopic	Transseptal	*p* Value	Microscopic	Transseptal	*p* Value	Microscopic	Transseptal	*p* Value
Physical functioning	92.7 ± 13.8	93.4 ± 6.97	0.828	84.8 ± 17.3	83.4 ± 15.6	0.767	87.8 ± 18.5	90 ± 9.03	0.627
Physical role functioning	74.3 ± 37.6	84.1 ± 37.4	0.341	39.3 ± 37.5	35.2 ± 32.4	0.689	77.5 ± 36.2	67.5 ± 37.3	0.395
Bodily pain	85.5 ± 16.8	76.7 ± 14.0	**0.047**	80.8 ± 13.6	78.2 ± 14.1	0.511	88.4 ± 16.0	83.5 ± 14.5	0.311
General health perceptions	69.3 ± 17.9	57.1 ± 18.3	**0.017**	70.8 ± 16.3	70.0 ± 17.1	0.856	73.3 ± 17.7	65.9 ± 19.7	0.216
Vitality	71.7 ± 17.4	71.6 ± 15.6	0.978	73.9 ± 13.9	68.6 ± 19.5	0.268	76.5 ± 12.3	70.5 ± 15.2	0.178
Social role functioning	94.6 ± 18.8	88.9 ± 23.1	0.310	90.6 ± 21.9	86.4 ± 25	0.524	99.4 ± 16	88.8 ± 25.9	0.127
Emotional role functioning	64.8 ± 44.2	66.7 ± 37.1	0.867	69 ± 41.5	65.2 ± 41.8	0.744	86.7 ± 27.4	76.7 ± 30.8	0.284
Mental health	73.6 ± 16.3	69.8 ± 16.4	0.398	77.7 ± 16.6	70.5 ± 19.8	0.206	75.2 ± 17.4	75 ± 13.4	0.968
Reported Health Transition	41.4 ± 17.1	46.6 ± 20.8	0.312	51.8 ± 26.3	52.3 ± 24.3	0.947	50 ± 26.9	62.5 ± 25	0.136
Total	124 ± 15.6	120 ± 12.2	0.361	122 ± 13.4	118 ± 16.2	0.365	127 ± 11.7	123 ± 12.9	0.332

Values represent mean± standard deviation.

Bold values: statistically significant.

### 3.3 Sino-Nasal Quality of Life Scales

No statistically significant difference in total SNOT-22 scores between the two groups was observed before surgery (**Table 3**). The transseptal group showed significantly lower total SNOT-22 scores than the microscopic group at 1 month postoperatively (*p* = 0.011); however, no significant difference was observed at 3 months postoperatively. Both groups showed a significant increase in SNOT-22 score from preoperation to 1 month postoperatively (microscopic group: 13.7 ± 3.11, *p* < 0.001; transseptal group: 11.3 ± 2.89, *p* < 0.001) and recovered by 3 months postoperatively (microscopic group: 2.06 ± 1.29; *p* = 0.174, transseptal group: 1.5 ± 0.76; *p* = 0.687).

**Table 3:** SNOT-22.

	Microscopic (n = 35)	Transseptal (n = 23)	*P* Value
Pre-operation	1.43 ± 1.07	1.39 ± 1.16	0.892
PO 1 month	13.7 ± 3.11	11.3 ± 2.89	**0.011**
PO 3 months	2.06 ± 1.29	1.5 ± 0.76	0.112

Values represent mean± standard deviation.

Bold values: statistically significant.

Both groups showed a significant change in ASK score at 1-month follow-up from preoperation (*p* < 0.001) but did not recover by 3 months postoperatively (*p <* 0.001; **Table 4**). Furthermore, the transseptal group showed lower ASK scores than those of the microscopic group 3 months postoperatively (*p* = 0.025).

**Table 4:** Anterior skull base nasal inventory survey.

	Microscopic (n = 35)	Transseptal (n = 23)	*P* Value
Pre-operation	2.21 ± 1.04	1.96 ± 0.976	0.366
PO 1 month	11.4 ± 4.26	10.5 ± 5.41	0.523
PO 3 months	9.26 ± 4.71	6.29 ± 3.30	**0.025**

Values represent mean± standard deviation.

Bold values: statistically significant.

### 3.4 Olfactory Functions

Subjective olfactory function based on VAS score showed a marked decrease 1 month after surgery (microscopic group: 7.17 ± 1.76 vs 9.43 ± 1.40, *p* = 0.002; transseptal group: 6.50 ± 1.77 vs 9.88 ± 0.342, *p* = 0.001) in both groups (**Table 5**). Only the microscopic group fully recovered olfactory function to preoperative scores at 3 months postoperative follow-up (microscopic group: 8.93 ± 0.832 vs 9.43 ± 1.40, *p* = 0.092; transseptal group: 8.91 ± 0.970 vs 9.88 ± 0.342, *p* = 0.003). No significance difference in VAS score was found between the two groups in preoperatively (*p* = 0.22) or at 1-month (*p* = 0.29) or 3-months follow-up *(p* = 0.95).

**Table 5:** Olfactory function based on VAS.

	Preoperative			PO 1 Month			PO 3 Months		
	Microscopic	Transseptal	*p* Value	Microscopic	Transseptal	*p* Value	Microscopic	Transseptal	*p* Value
VAS	9.43 ± 1.40	9.88 ± 0.342	0.222	7.17 ± 1.76	6.50 ± 1.77	0.291	8.93 ± 0.832	8.91 ± 0.970	0.951

Values represent mean± standard deviation.

According to the Sniffin’ Sticks test, in the microscopic group, five cases (14%) were defined as hyposmic 1 month after surgery, and two cases (6%) were defined as hyposmic 3 months after surgery (**Table 6**). In contrast, one case (4%) was defined as hyposmic 1 and 3 months after surgery in the transseptal group. No anosmic patients were found in either the microscopic or transseptal group. No significant difference was observed for total TDI scores among preoperative and 1 and 3 months postoperative scores in either group (**Table 7**). Similarly, OT, OD, and OI scores showed no significant differences among time points in either group.

**Table 6:** Olfactory function based on the Sniffin’ Sticks test.

	PO 1 Month			PO 3 Months		
	Microscopic	Transseptal	*p* Value	Microscopic	Transseptal	*p* Value
Normal	30	22	0.498	33	22	0.818
Hyposmics	5	1	0.224	2	1	0.818
Anosmics	0	0	NA	0	0	NA

NA, Not applicable.

**Table 7:** TDI scores of the Sniffin’ Sticks test.

	Preoperative			PO 1 Month			PO 3 Months		
	Microscopic	Transseptal	*p* Value	Microscopic	Transseptal	*p* Value	Microscopic	Transseptal	*p* Value
OT	8.96 ± 3.16	8.64 ± 3.39	0.759	8.79 ± 2.36	8.97 ± 3.33	0.845	9.20 ± 1.90	9.51 ± 2.97	0.692
OD	13.0 ± 1.72	11.8 ± 2.88	0.111	12.5 ± 2.61	11.1 ± 3.77	0.167	12.4 ± 2.24	12.2 ± 2.96	0.769
OI	12.0 ± 1.68	12.1 ± 2.36	0.930	11.9 ± 1.68	11.5 ± 2.83	0.595	12.0 ± 1.02	11.9 ± 2.11	0.829
TDI	34.1 ± 4.16	32.5 ± 5.94	0.318	33.2 ± 4.47	31.7 ± 8.49	0.470	33.6 ± 3.39	33.5 ± 6.35	0.916

### 3.5 Post-Operative Nasal Endoscopy Outcomes

**Figure 3:**
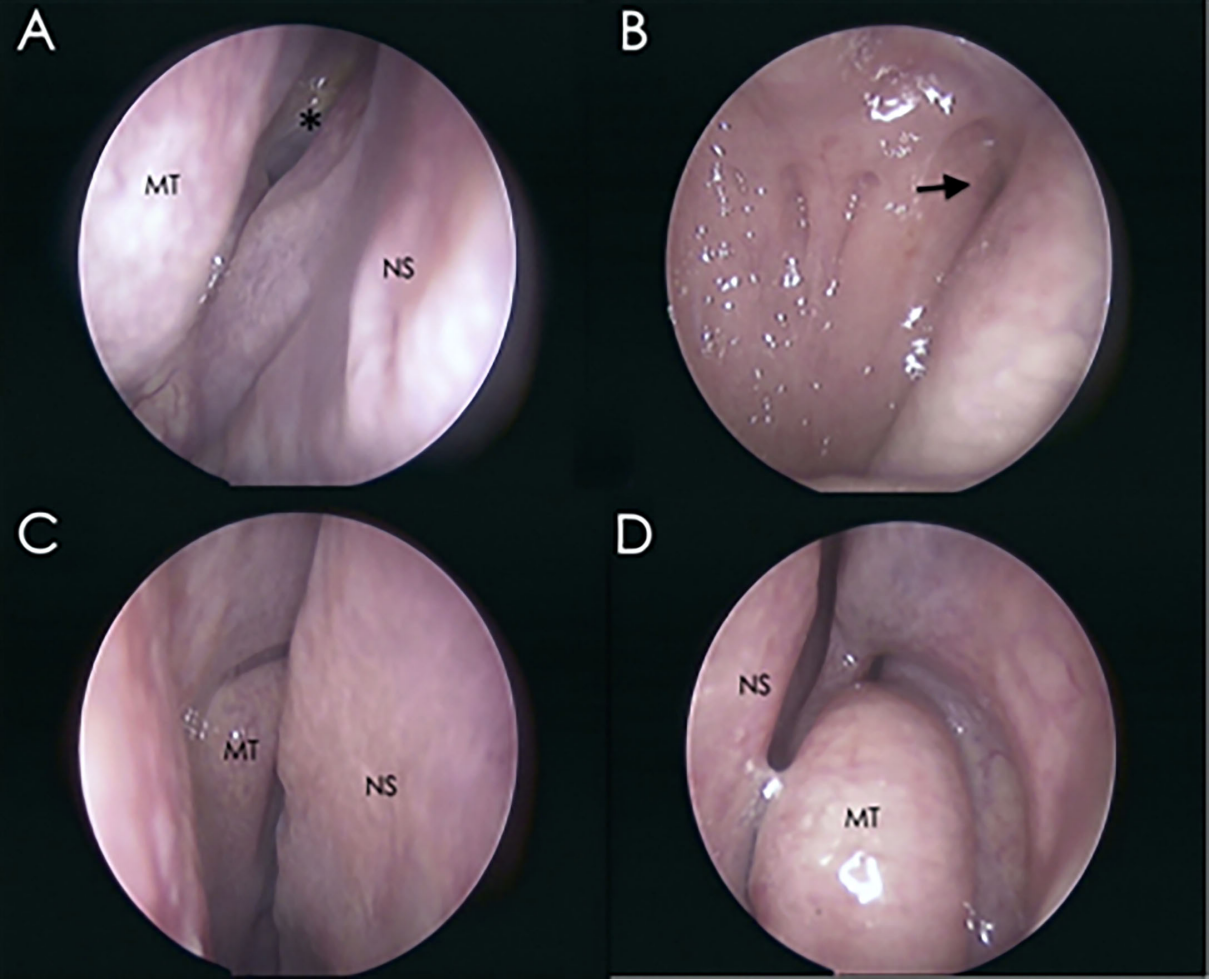
Representative nasal endoscopic images of outpatients at 1-month follow-up after the operation. Images show the integrity of the nasal structure. **(A)** Endoscopic view of the sphenoid ostium from the right nostril. **(B)** View of the nasopharynx. **(C)** Endoscopic view from the right nostril. **(D)** Endoscopic view from the left nostril. Asterisk, sphenoid ostium; arrow, pharyngeal recess; MT, middle turbinate; NS, nasal septum.

All patients were followed up using nasal endoscopy in the outpatient department at 1 and 3 months postoperatively. No significant differences were observed for the following outcomes: nasal septum perforation, nasal scabs, nasal congestion, nasal ostium obstruction, hypertrophy of the nasal turbinate, or deviated nasal septum between the two groups (**Table 8** and **Figure 3**).

**Table 8:** Nasal endoscopy outcomes.

Symptom	PO 1 Month			PO 3 Months		
	Microscopic	Transseptal	p Value	Microscopic	Transseptal	*p* Value
Nasal septum perforation	4	4	0.519	2	2	0.661
Nasal scabs	12	8	0.969	2	1	0.837
Nasal congestion	12	6	0.509	6	4	0.980
Nasal ostium obstruction	8	6	0.779	0	1	0.213
Hypertrophy of nasal turbinate	8	7	0.519	6	5	0.662
Deviated nasal septum	8	5	0.920	2	4	0.153

### 3.6 Complications

No carotid artery injury, intracerebral hemorrhage, epistaxis, meningitis, or visual worsening occurred in either group (**Table 9**). Postoperative nasal hemorrhage and unplanned second surgery rates were not significantly differently distributed between the two approaches. We observed a significantly higher incidence of intraoperative cerebrospinal fluid (CSF) leakage in the transseptal group than in the microscopic group (*p* = 0.013). Complication results are summarized in **Table 8**.

**Table 9:** Complications.

Variable	Microscopic (n=35)	Transseptal (n=23)	*P* Value
Carotid artery injury	0	0	>0.99
Intracerebral hemorrhage	0	0	>0.99
Epistaxis	0	0	>0.99
Meningitis	0	0	>0.99
Visual worsening	0	0	>0.99
Intraoperative CSF leakage	5	10	**0.013**
Postoperative CSF leakage	1	1	0.761
Postoperative nasal hemorrhage	2	1	0.818
Unplanned 2nd surgery	2	1	0.818

Bold values, statistically significant.

## 4 Discussion

The transnasal endoscopic route for pituitary surgery has been a favorable access route that has numerous advantages over microscopic surgery for large invasive cases. However, some studies have reported a negative impact on short-term olfaction and nasal QoL. A recent meta-analysis revealed a similar result on postoperative olfaction outcomes, although heterogeneity across the included studies was high (I^2^ > 95%, *p* <.01), which suggested significant variation in the included studies (9).

The purpose of this study was to introduce a novel modified microscopic-endoscopic bilateral transseptal approach and compare sinonasal QoL, olfactory function, and nasal endoscopy outcomes with those of the traditional microscopic approach. We hypothesized that this modified approach would be associated with similar nasal outcomes because it theoretically allows the preservation of almost all nasal mucosa and nasal structures.

### 4.1 Characteristics of the Modified Microscopic-Endoscopic Bilateral Transseptal Approach

The advantages of the modified microscopic-endoscopic bilateral transseptal approach are:

1. Bilateral nasalseptal mucosa flaps can be protected and left intact after the operation.
2. All surgical procedures can be performed by one person before tumor resection.
3. Surgical exposure is similar to that of the standard binostril endoscopic approach.
4. All surgical instruments are limited to the corridor between the two nasalseptal mucosa flaps, which makes it easier to achieve aseptic status. When intraoperative CSF leakage occurs, it is important to maintain an aseptic status to avoid severe central nervous system infection.
5. Final inspection of the surgical field after tumor resection can be performed quickly and easily. The frequency of electrocoagulation hemostasis is much lower than that of the standard endoscopic approach.
6. Each nasalseptal mucosa flap is theoretically large enough to be used as a pedicled nasal mucosal flap to reconstruct the skull base in larger extensive surgery.
7. The resected nasalseptal bone can be inserted and repositioned between the bilateral nasalseptal mucosa flaps.

The disadvantages and limitations of the modified microscopic-endoscopic bilateral transseptal approach are:

1. In cases of narrow nasal space, more attention needs to be paid to the dissection of the whole nasalseptal mucosa flap to minimize surgery-related injury.
2. Lateral exposure is limited by the narrow anterior wall of the sphenoidal sinus and bilateral superior turbinate.
3. Because bilateral mucosa linear incision is near the nasal vestibule, postoperative acute bleeding must be prevented.
4. In recurrent cases, the dissection of the bilateral nasalseptal mucosa flap is difficult.

We recommend the following surgical technique tips:

1. Bilateral hemitransfixion incisions on the septal mucosa should not be the same depth in case of postoperative nasal septal perforation.
2. Infiltration of the epinephrine solution should be performed after the dissection of the bilateral septal mucosa flaps. Because the mucosa flap will be vasoconstricted and thinner, it will be vulnerable to becoming lacerated during the subperiosteal dissection of the septal flap.
3. The anterior sphenoidal wall should be removed as much as possible under microscopy by one neurosurgeon.

According to our experiences on the modified microscopic-endoscopic bilateral transseptal approach, it might be not appropriate for those who need nasal mucosa flap to repair CSF leakage. Thus, further studies are required to optimize this approach. Another limitation of this approach is re-transnasal surgery, because the septal mucosa flap is easier to be broken during dissection. The standard endoscopic approach would be more appropriate for recurrent pituitary adenomas.

### 4.2 Clinical Outcomes

In this study, the transseptal approach was demonstrated to be an effective and safe transsphenoidal approach for pituitary adenoma resection. Although the tumors were significantly larger in the transseptal group, the same surgical effect was achieved in terms of GTR rate and surgery-related complications, which included artery injury, intracerebral hemorrhage, meningitis, visual worsening, postoperative CSF leakage, and epistaxis. In the transseptal group, we observed a higher incidence of intraoperative CSF leak due to aggressive resection of the larger tumor using endoscopic instruments. However, the incidence of postoperative CSF leak during the transseptal approach was not significantly higher than that during the microscopic approach, which demonstrated that the intraoperative repair of CSF rhinorrhea under the transseptal approach is reliable.

### 4.3 Quality of Life

The SF-36 test to evaluate the postoperative QoL of patients showed that of the eight subdomains, the PF and PR subdomain scores decreased at 1-month follow-up in both groups; however, full recovery at 3 months postoperatively was only achieved in the microscopic group, which indicated that the microscopic approach could enable patients to be physically ready for regular work and activities in 3 months. In contrast, the transseptal approach may require more than 3 months to achieve full recovery of PF function; although postoperative GH significantly improved following surgery, which was not observed in the microscopic group. This may be attributed to the larger tumor volume and higher incidence of CSF leak in the transseptal group. Because of the larger tumor size, the transseptal group patients experienced more complaints and clinical syndromes than did the microscopic group. Thus, preoperative GH subdomain scores were much lower in the transseptal group than in the microscopic group. After tumor resection, both groups’ complaints and clinical syndromes significantly improved, which resulted in a significant difference in the GH subdomain score. During preoperative instructions of the surgery, patients in both groups were routinely informed of the size of the pituitary tumor and the possible complications during surgery. As a result, the transseptal group had a larger tumor size and higher occurrence of intraoperative CSF leak. Although the repair of the sellar floor was successful, we still strongly recommended patients who had intraoperative CSF leak to remain home to rest and avoid any heavy work or strenuous exercise following discharge. Such preoperative instruction and postoperative education may have influenced the PF subdomain scores at the 3-month follow-up. These patients likely required more time to convince themselves that they are of sufficient health and ready to return to work.

### 4.4 Nasal Outcome

Postoperative endoscopic inspection revealed no differences between the two groups for nasal septum perforation, nasal scabs, nasal congestion, nasal ostium obstruction, hypertrophy of the nasal turbinate, or deviated nasal septum. Three months after the operation, the nasal structure of most patients returned to the preoperative level (**Table 7**).

At 1 month postsurgery, patients in both groups showed significantly higher SNOT-22 and ASK scores. However, the scores of the transseptal group were significantly lower than those of the microscopic group, which was not expected. At 3 months postoperatively, SNOT-22, but not ASK, scores had fully recovered in both groups, and neither SNOT-22 nor ASK scores were significantly different between the two groups. This demonstrated that surgery-related nasal QoL decreased 1 month postoperatively, and more than 3 months were needed to achieve full recovery of nasal QoL. The transseptal approach was slightly better than the microscopic approach in the early stage of recovery.

The subjective olfactory VAS test showed that the scores of both groups significantly decreased 1 month after surgery, but full recovery after 3 months was achieved in only the microscopic group. However, the semi-objective olfactory test results did not change preoperatively to 1 month after surgery in either group, and no differences were found between groups at 1- or 3-months follow-ups. These contradicted results between the subjective olfactory VAS test and the semi-objective Sniffin’ Sticks test are intersesting. This suggests that subjective evaluations, such as the VAS test, may not provide an accurate assessment of olfactory function due to patients’ subjective scoring. Thus, the Sniffin’ Sticks test, a validated psychophysical tool allowing detailed, semi-objective evaluation of a patient’s olfactory performance, is recommended for accurate evaluations.

Taken together, our results indicated that the transseptal approach enables the preservation of nasal QoL, olfactory function, and nasal structure to the levels achieved by the microscopic approach. Olfactory dysfunction, which often presents as hyposmia or anosmia, and a decrease in nasal QoL leads to a profound decrease in QoL and mental health and an increase in depression. A pituitary adenoma is a benign lesion that can be controlled or cured long-term by total or partial removal of the tumor. Thus, neurosurgeons should consider preservation of QoL as one of the primary objectives of surgery. However, a paradigm shift in recent decades has placed greater importance on patients’ functional outcomes, which has prompted investigations on olfactory function and nasal QoL after transsphenoidal surgery for pituitary adenoma. We modified a previously implemented transseptal approach to achieve not only the same protection of nasal mucosa as that of microscopic surgery but also the same surgical effects as endoscopic pituitary adenoma surgery. Moreover, we compared this modified transseptal approach with the microscopic transnasal approach in terms of olfactory outcomes, nasal QoL, QoL, and postoperative nasal structure. The overall outcome of this new modified approach was largely positive, as expected.

### 4.5 Limitations

Several limitations of this study should be considered. Although the sample size met the criteria of the power calculation, preoperative SF-36 scores for BP and GH components were poorer in the transseptal group compared with those of the microscopic group. Furthermore, patients in the transseptal group had larger pituitary tumor volumes compared with those of the transnasal group. A larger sample size may allow further comparisons with standard transnasal endoscopic approaches or even extended skull base surgery. Moreover, a longer follow-up period will enable additional and longer-term characterization of changes in olfactory outcomes. Future large-scale prospective or randomized controlled trials are needed to verify these findings.

## 5 Conclusions

The modified microscopic-endoscopic bilateral transseptal approach provides similar postoperative nasal QoL, olfactory function, nasal structure, and QoL as does the microscopic approach. Olfactory function recovered to preoperative levels 1 month after surgery following either approach. Full recovery of nasal and general QoL requires at least 3 months. The transseptal approach resulted in similar olfactory outcomes as those achieved using the uninostril microscopic approach but without any loss of endoscopic surgical vision, combined with the advantages of both the microscopic and endoscopic approaches. This modified approach has the potential to become the optimal endoscopic approach for pituitary adenoma surgery.

## Data Availability Statement

The raw data supporting the conclusions of this article will be made available by the authors, without undue reservation.

## Ethics Statement

The studies involving human participants were reviewed and approved by The Institutional Ethics Committee of Huashan hospital Fudan University. The patients/participants provided their written informed consent to participate in this study. Written informed consent was obtained from the individual(s) for the publication of any potentially identifiable images or data included in this article.

## Author Contributions

CS and BY conceived of the presented idea. JJZ developed the theoretical formalism, performed the anlytic calculations. YG and JJZ performed the surverys. BY supervised the project. All authors discussed the results and contributed to the final verion of the manuscript.

## Funding

This work was supported by grants (82001140) from The National Nature Science Foundation and Shanghai Municipal Government, and also supported by CAMS Innovation Fund for Medical Sciences (CIFMS, 2019-I2M-5-008).

## Conflict of Interest

The authors declare that the research was conducted in the absence of any commercial or financial relationships that could be construed as a potential conflict of interest.

The handling editor declared a shared parent affiliation with the authors at time of review.

## Publisher’s Note

All claims expressed in this article are solely those of the authors and do not necessarily represent those of their affiliated organizations, or those of the publisher, the editors and the reviewers. Any product that may be evaluated in this article, or claim that may be made by its manufacturer, is not guaranteed or endorsed by the publisher.
